# A Train Factor Graph Fusion Localization Method Assisted by GRU-IBiLSTM for Low-Cost SINS/GNSS

**DOI:** 10.3390/s26041226

**Published:** 2026-02-13

**Authors:** Cheng Chen, Guangwu Chen, Xinye Ma

**Affiliations:** 1 School of Electronic and Information Engineering, Lanzhou Jiaotong University, Lanzhou 730070, China; 11230765@stu.lzjtu.edu.cn; 2 School of Automation and Electrical Engineering, Lanzhou Jiaotong University, Lanzhou 730070, China

**Keywords:** train integrated positioning, factor graph, GRU-IBiLSTM, trajectory forecasting

## Abstract

The integrated strapdown inertial navigation system (SINS)/global navigation satellite system (GNSS) has been widely adopted in railway positioning applications. However, conventional filtering-based approaches are fundamentally constrained by their dependence on instantaneous state estimates while failing to exploit valuable historical measurement information. To overcome this limitation, we develop a factor graph optimization (FGO) framework to enhance data utilization efficiency. During GNSS signal outages, existing implementations typically preserve only SINS factors while excluding GNSS observations, leading to unbounded error growth. To bridge this gap, our novel solution integrates a gated recurrent unit (GRU) with an Improved Bidirectional Long Short-Term Memory (IBiLSTM) network to generate accurate pseudo-GNSS observations through effective learning from both preceding and subsequent GNSS data sequences. Comprehensive evaluation under GNSS-denied conditions demonstrates that our approach achieves significant improvements over conventional neural network-aided methods, with horizontal root mean square error (RMSE) reductions of 49.22% (simulation) and 36.24% (onboard vehicle). Subsequent FGO processing yields additional performance gains, further reducing RMSE by 46.67% (simulation) and 35.31% (onboard vehicle). This innovative methodology effectively maintains positioning accuracy and ensures navigation continuity during GNSS outages, thereby offering a robust solution for train positioning systems in challenging environments.

## 1. Introduction

The safe operation of trains is contingent upon precise onboard positioning technology [1]. The positioning approach based on the SINS/GNSS integrated navigation solution has been extensively utilized in train positioning systems [2,3].

At present, data fusion methods for SINS/GNSS integrated navigation can be classified into two main categories, namely filter-based approaches and optimization-based approaches [4]. Among these, filter-based methods are the most extensively applied, with the Kalman Filter (KF) [5] being particularly prominent due to its state-space formulation that enables optimal estimation of system states by integrating measurement data [6]. Nevertheless, the conventional Kalman Filter exhibits inherent limitations, including its reliance on linear system models and the assumption of stationary Gaussian observation noise. To overcome these constraints, researchers have proposed several enhanced variants, such as the Extended Kalman Filter (EKF) for nonlinear systems [7], the Unscented Kalman Filter (UKF) [8], and the Cubature Kalman Filter (CKF) [9]. Despite these improvements, a core limitation remains in that these methods rely solely on the most recent state estimate and current observations, resulting in an inefficient use of historical information [10].

In contrast to filter-based recursive estimation, optimization-based smoothing methods possess a stronger capability to jointly exploit a span of historical information within a unified framework. A representative technique in this category is the factor graph (FG) [11], which represents the probabilistic relationships between system states and multi-source measurements using an undirected graphical model and solves for the states through maximum a posteriori (MAP) estimation [11,12]. Under common assumptions such as conditional independence of measurements and Gaussian noise, the estimation problem can be reformulated as the minimization of a global residual cost function, thereby enabling the unified fusion of heterogeneous, multiple rate, and asynchronous measurements [12,13]. Owing to its globally consistent smoothing formulation and joint optimization over all states, the FG has attracted increasing attention in navigation systems integrated with SINS in recent years. Compared with traditional filtering approaches, incremental smoothing in factor graphs preserves historical states for joint optimization and relinearizes affected variables when new information arrives, achieving accuracy close to that of global smoothing while simultaneously reducing computational burden and mitigating the suboptimality and inconsistency issues caused by marginalization in filtering-based methods [14]. Building on this foundation, Song et al. [15] incorporated factor graphs into a multi-source integrated navigation system to better tackle challenges such as sensor asynchrony and information latency. Chiu et al. [16] further enhanced the methodology by integrating a sliding-window mechanism into the navigation algorithm. This approach restricts the factor graph’s global optimization to the most recent windowed factor nodes, thereby enhancing computational efficiency without compromising estimation accuracy. Subsequently, Li et al. [17] demonstrated through simulations that factor graphs significantly optimize GNSS/INS state estimation by effectively utilizing historical data. For practical validation, Xu et al. [18] realized reliable vehicle positioning using SINS/GNSS FGO, while Mu et al. [19] capitalized on the low-cost and highly autonomous characteristics of SINS to develop a SINS/GNSS integrated navigation system based on FG. However, during actual train operations, frequent transitions through complex environments such as forests, tunnels, and culverts often induce GNSS signal outages. Under such conditions, the absence of sufficient measurement constraints results in the accumulation of SINS positioning errors over time, ultimately inducing progressive divergence [20]. Consequently, the application of FGO to fuse SINS data and sustain positioning accuracy during GNSS-denied periods has emerged as a critical research focus in integrated navigation [21].

Chun et al. [22] and Vavilova et al. [23] developed an integrated navigation system that combines SINS, GNSS, and odometer (ODO) sensors. Li et al. [24] and Reimer et al. [25] extended this framework by incorporating MEMS sensors to construct a MEMS/INS/ODO/GNSS multi-source fusion architecture. These multi-sensor configurations enhance positioning accuracy and robustness; however, they also increase system complexity and cost. In parallel, the rapid advancement of artificial intelligence has spurred the development of neural network-based methods to mitigate GNSS signal outages [26,27]. Such methods leverage trained neural networks to predict pseudo-GNSS observations, thereby compensating for positioning errors during signal interruptions [28]. Dai et al. [29] applied a Recurrent Neural Network (RNN) to improve INS/GNSS positioning performance under GNSS-denied conditions [21]. However, RNNs suffer from overfitting, vanishing gradients, and exploding gradients, which limit their effectiveness. To overcome these issues, improved RNN variants have been developed, such as the GRU adopted by Xu et al. [30] to model SINS error dynamics and perform real-time compensation during outages, effectively suppressing error accumulation and enhancing system robustness. Similarly, Zhao et al. [31] utilized Long Short-Term Memory (LSTM) networks to predict pseudo-GNSS observations and enhance positioning accuracy during signal loss. Despite their advantages, both GRU and LSTM rely on unidirectional structures, which restrict their ability to capture bidirectional temporal dependencies. Addressing this, Xu et al. [18] addressed this limitation by introducing a Bidirectional LSTM (BiLSTM) model that learns temporal features from both past and future time steps, thereby improving prediction accuracy. Researchers have also explored other time-series forecasting methods, including Autoregressive Integrated Moving Average (ARIMA) [21], and hybrid neural architectures such as CNN-GRU [32], CNN-LSTM [33], and GRU-LSTM [34].

Inspired by the aforementioned studies, this paper proposes a trainable factor graph-based fusion positioning method for trains to address positioning failures caused by GNSS signal outages. Specifically, a GRU-IBiLSTM temporal prediction model is developed to learn from historical GNSS measurements and generate pseudo-GNSS observations during GNSS interruptions, thereby compensating for missing measurement information. The generated pseudo-GNSS observations, together with SINS measurements, are integrated into the FG framework for smoothing-based optimization, which enables the SINS/GNSS integrated navigation system to maintain high-precision positioning performance under prolonged signal loss. To validate the proposed method, experiments are conducted in a tunnel environment, a representative scenario of GNSS signal blockage, and the results demonstrate the method’s effectiveness, adaptability, and improved positioning accuracy. The main contributions of this work are summarized as follows:

(1) A time-series forecasting model incorporating GRU-IBiLSTM architectures is proposed to generate pseudo-GNSS observations during signal interruptions. By compensating for outages with high-precision predictions, the model ensures uninterrupted and reliable operation of the SINS/GNSS integrated train positioning system.
(2) A probabilistic model structured on factor graphs is employed for SINS/GNSS data fusion. In contrast to conventional filtering methods, the proposed model achieves a more comprehensive utilization of historical information, leading to a substantial improvement in navigation accuracy.
(3) An innovative framework integrating the factor graph probabilistic model with the GRU-IBiLSTM temporal predictor is presented. This cross-domain fusion of pseudo-measurements yields a hardware-free, low-cost solution, significantly improving the continuity and robustness of low-cost SINS/GNSS train positioning systems.

This paper is organized as follows. Chapter 2 analyzes the architecture of the proposed SINS/GNSS integrated navigation system for trains. Chapter 3 presents a low-cost fusion algorithm for SINS/GNSS train positioning based on the FGO. Chapter 4 details the GRU-IBiLSTM forecasting model used to generate pseudo-GNSS observations. Chapter 5 provides experimental results to validate the effectiveness of the proposed method. Chapter 6 concludes the paper and summarizes the key contributions.

## 2. Overview of the Proposed Navigation System

**Figure 1:**
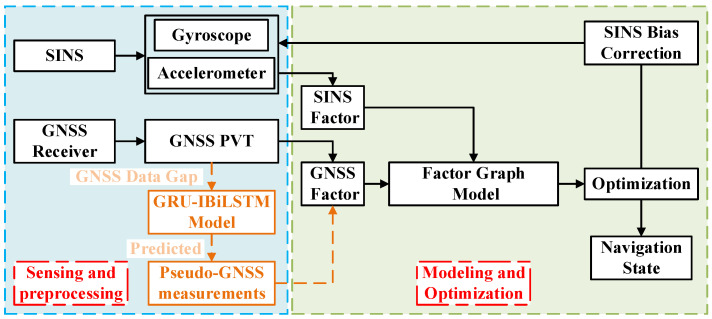
Flowchart of the low-cost SINS/GNSS integrated navigation system aided by the GRU-IBiLSTM network.

This paper addresses the challenge of GNSS signal outages in low-cost SINS/GNSS integrated train navigation systems operating in complex railway environments, proposing a robust fusion algorithm based on FGO. As illustrated in Figure 1, SINS takes measurements from a low-cost system consisting of a triaxial gyroscope and a triaxial accelerometer as input and recursively propagates the inertial mechanization equations to produce high-rate navigation state estimates of attitude, velocity, and position. These SINS outputs provide high short-term accuracy but gradually drift due to the accumulation of inertial sensor biases and integrated noise. GNSS provides globally referenced positioning observations with long-term stability, yet its signals are vulnerable to blockage, interference, and multipath effects in railway scenarios such as tunnels, stations, and cuttings, which leads to intermittent measurement outages. By integrating the two systems, GNSS provides long-term constraints that limit the drift of the SINS, while the high-frequency SINS navigation state outputs bridge the low-rate and intermittent GNSS updates, thereby achieving complementary performance.

However, during prolonged GNSS outages, navigation errors tend to accumulate and diverge due to the reliance solely on SINS constraints. To proactively address this challenge, this paper proposes a hybrid enhancement method. The core of this method lies in leveraging FGO to globally refine the SINS/GNSS integrated train’s positioning system under normal GNSS signal conditions. Simultaneously, GNSS measurements are utilized to train the sequential forecasting model, GRU-IBiLSTM. In the event of GNSS outages, the FG framework integrates pseudo-GNSS observations generated by the GRU-IBiLSTM model, integrated with SINS solutions, thereby effectively mitigating the divergence of pure inertial navigation errors. This hybrid architecture fully leverages the global optimization capability of the factor graph and the sequential forecasting capability of GRU-IBiLSTM, ensuring continuous and robust positioning performance, even under complex train operating scenarios.

## 3. Low-Cost SINS/GNSS Integration Based on Factor Graph

This chapter presents a factor graph-based modeling and optimization framework for low-cost SINS/GNSS integrated navigation. Section 3.1 introduces the construction principles of the FG model, including the definitions of nodes and constraint relationships. Section 3.2 provides a detailed explanation of the formulation of SINS factors derived from inertial measurements and pre-integration. Section 3.3 describes the modeling of prior and GNSS factors. Section 3.4 outlines the complete FGO framework.

### 3.1. Factor Graph Model

The FG model approach decomposes a global multivariate function into a product of local factor functions, which effectively characterize the joint probability dependencies among random variables [11]. This relationship can be expressed as:

(1) $$G=\left(F,X,E\right),$$

In Equation (1), F represents the set of factor nodes, decomposed into local functions $f_{i}\in F$. X denotes the set of variable nodes, while $x_{j}\in X$ corresponds to the variables in the global multivariate function. E defines the set of connecting edges, which establishes the linkage between the factor node $f_{i}$ and the variable node $x_{j}$ via the connecting edge $e_{ij}\in E$, yielding:

(2) $$f\left(x_{1},x_{2},\cdots \cdots ,x_{m}\right)=f_{1}\left(X_{1}\right)f_{2}\left(X_{2}\right)\cdots \cdots f_{n}\left(X_{n}\right),$$

where m denotes the number of variable nodes, n represents the number of independent variable node sets, and $X_{i}$ indicates the set of all variable nodes associated with the factor node $f_{i}$, with $X_{i}\subseteq X$.

By combining Equations (1) and (2) and rewriting the factorization in a compact and equivalent notation using $f\left(X\right)$ and the product operator ∏, the FG method is applied to perform factorization. This is equivalently expressed as Equation (3):

(3) $$f\left(X\right)=\prod_{i}f_{i}\left(X_{i}\right),$$

Let the variable node $x_{i}\in {\mathbb{R}}^{15}$ represent the train positioning state at time $t_{i}$, and denote the state history from $t_{0}$ to $t_{k}$ as $X_{k}=\left\{x_{0},x_{1},\cdots \cdots ,x_{k}\right\}$. Assume $Z_{i}=[Z_{i}^{\mathrm{SINS}},Z_{i}^{\mathrm{GNSS}}]\in {\mathbb{R}}^{12}$ represents the measurement data from the SINS and GNSS at $t_{i}$ time, where $Z_{i}^{\mathrm{SINS}}\in {\mathbb{R}}^{6}$ and $Z_{i}^{\mathrm{GNSS}}\in {\mathbb{R}}^{12}$. The measurement data from SINS includes the raw data from the accelerometer and the gyroscope, which provide the accelerations along the three axes and the angular velocities, respectively. The measurement data from GNSS refers to the position and velocity solutions provided by the GNSS receiver, which can be written as $Z_{i}^{\mathrm{GNSS}}=\left[p_{i}^{\mathrm{GNSS}},v_{i}^{\mathrm{GNSS}}\right]$, where $p_{i}^{\mathrm{GNSS}}\in {\mathbb{R}}^{3}$ and $v_{i}^{\mathrm{GNSS}}\in {\mathbb{R}}^{3}$. Define the measurement history from $t_{0}$ to $t_{k}$ as $Z_{k}=\left\{z_{0},z_{1},\cdots \cdots ,z_{k}\right\}$. Based on the prior probability $P(X_{k})$ of state $X_{k}$ and incorporating Bayes’ theorem (4):

(4) $$P\left(A|B\right)=\frac{P\left(A\right)P\left(B|A\right)}{P\left(B\right)},$$

The state and measurement information of the SINS/GNSS train integrated positioning system can be expressed as a joint probability density function, as shown in (5):

(5) $$P\left(X_{k}|Z_{k}\right)=\frac{P\left(X_{k}\right)P\left(Z_{k}|X_{k}\right)}{P\left(Z_{k}\right)}\propto P\left(X_{k}\right)P\left(Z_{k}|X_{k}\right),$$

The MAP estimation of (5) is obtained by maximizing $P\left(X_{k}|Z_{k}\right)$, yielding (6):

(6) $$\begin{array}{ll} X_{k}^{\mathrm{MAP}} & =\underset{X_{k}}{\mathrm{argmax}}\;P\left(X_{k}|Z_{k}\right)\\ & =\underset{X_{k}}{\mathrm{argmax}}\left[P\left(X_{k}\right)\frac{P\left(Z_{k}|X_{k}\right)}{P\left(Z_{k}\right)}\right]\\ & \propto \underset{X_{k}}{\mathrm{argmax}}\left[P\left(X_{k}\right)P\left(Z_{k}|X_{k}\right)\right] \end{array},$$

The probability distribution of the optimal localization solution, conditioned on the measurement set and the estimated state set, can be factorized from (6), yielding (7):

(7) $$\begin{array}{ll} X_{k}^{\mathrm{MAP}} & \propto \underset{X_{k}}{\mathrm{argmax}}\left[P\left(X_{k}\right)P\left(Z_{k}|X_{k}\right)\right]\\ & \propto P(x_{0})\prod_{i=1}^{k}[P\left(x_{i}|x_{i-1},\alpha_{i-1},z_{i-1}^{\mathrm{SINS}}\right)P\left(\alpha_{i}|\alpha_{i-1}\right)\prod_{z_{j}\in Z_{i}/z_{i}^{\mathrm{SINS}}}P\left(z_{j}|x_{i}^{j}\right)] \end{array},$$

where $P\left(x_{0}\right)$ represents the prior information of the system’s initial state. The term $P\left(\alpha_{i}|\alpha_{i-1}\right)$ denotes the conditional probability density of the SINS bias, and $P\left(x_{i}|x_{i-1},\alpha_{i-1},z_{i}^{\mathrm{SINS}}\right)$ denotes the posterior probability density of the system state. The parameter $\alpha_{i}$ represents the bias correction value of the SINS at time i, and $z_{i}^{\mathrm{SINS}}$ is the measurement from SINS at time i. The term $\prod_{z_{j}\in Z_{i}/z_{i}^{\mathrm{SINS}}}P\left(z_{j}|x_{i}^{j}\right)$ denotes the joint posterior probability density of GNSS measurements at time i, conditioned on the state $x_{i}^{j}$. In this formulation, $Z_{i}$ represents the complete set of sensor observations at time i, while $z_{i}^{\mathrm{SINS}}$ refers specifically to the measurements obtained from the SINS. Consequently, the expression $Z_{i}/z_{i}^{\mathrm{SINS}}$ identifies the subset of observations excluding those from SINS, namely the GNSS data. Each $z_{j}$ within this subset corresponds to an individual GNSS measurement, and $P\left(z_{j}|x_{i}^{j}\right)$ characterizes the likelihood of observing $z_{j}$ given the state $x_{i}^{j}$.

The optimal state estimate $X_{k}^{\mathrm{MAP}}$ for the state variable $X_{k}$ is obtained when the factor function $f\left(X\right)$ reaches its maximum, as expressed in Equation (8):

(8) $$X_{k}^{\mathrm{MAP}}=\underset{X_{k}}{\mathrm{argmax}\;}P\left(X_{k}|Z_{k}\right)\propto \underset{X_{k}}{\mathrm{argmax}}\prod_{i}f_{i}\left(X_{i}\right),$$

Accordingly, the joint probability density function can be expressed as a product of individual factors, given by (9):

(9) $$P\left(X_{k}|Z_{k}\right)\propto \prod_{i}f_{i}\left(X_{i}\right),$$

Each factor node is associated with an error function, defined in Equation (10) as:

(10) $$f_{i}\left(x_{i}\right)=d\left(h_{i}\left(x_{i}\right)-z_{i}\right),$$

where d(⋅) denotes the corresponding cost function, $h_{i}\left(x_{i}\right)-z_{i}$ represents the error function, $h_{i}(\cdot )$ represents the system measurement function, and $z_{i}$ corresponds to the actual sensor measurement.

Under the assumption of Gaussian noise, the factor node $f_{i}$ is formulated based on the error function as shown in Equation (11):

(11) $$d\left(h_{i}\left(x_{i}\right)-z_{i}\right)=e^{-\frac{1}{2}||h_{i}\left(x_{i}\right)-z_{i}|{|}_{\sum }^{2}},$$

where $||a|{|}_{\sum }^{2}=a^{T}\sum^{-1}a$ represents the squared Mahalanobis distance and ∑ is the measurement noise covariance matrix.

The system states and constraints across all time steps are formulated as a nonlinear least squares problem. By taking the negative natural logarithm of Equation (9) and incorporating Equations (10) and (11) into the resulting expression, the optimization objective is derived as:

(12) $$\begin{array}{l} X_{k}^{\mathrm{MAP}}\propto \underset{X_{k}}{\mathrm{argmax}}\prod_{i}f_{i}\left(X_{i}\right)\\ \;\;\;\;\;\;\;\;=\underset{X_{k}}{\mathrm{argmin}}\left(-\ln\prod_{i}f_{i}\left(X_{i}\right)\right)\\ \;\;\;\;\;\;\;\;=\underset{X_{k}}{\mathrm{argmin}}\sum_{i}||h_{i}\left(x_{i}\right)-z_{i}|{|}_{\sum }^{2} \end{array},$$

### 3.2. SINS Factor

The following are the established acceleration model (13) and angular velocity model (14) for SINS:

(13) $${\hat{f}}_{t}^{b}=f_{t}^{b}+b_{f_{t}}+\varepsilon_{f},$$

(14) $${\hat{\omega }}_{t}^{b}=\omega_{t}^{b}+b_{\omega_{t}}+\varepsilon_{\omega },$$

In Equations (13) and (14), ${\hat{f}}_{t}^{b}$ and $f_{t}^{b}$ represent the measured and true values of the accelerometer in the body frame (b-frame), respectively, while $b_{f_{t}}$ and $\varepsilon_{f}$ denote the accelerometer bias and noise. Similarly, ${\hat{\omega }}_{t}^{b}$ and $\omega_{t}^{b}$ indicate the measured and true values of the angular velocity, with $b_{\omega_{t}}$ and $\varepsilon_{\omega }$ corresponding to the gyroscope bias and noise. Therefore, the inertial measurements can be expressed as the sum of the true quantities and the associated bias and noise terms. For convenience in subsequent state estimation, we absorb the bias and noise into the sensor bias states and define:

(15) $$\nabla_{f}=b_{f_{t}}+\varepsilon_{f},$$

(16) $$\nabla_{\omega }=b_{\omega_{t}}+\varepsilon_{\omega },$$

where $\nabla_{f}$ and $\nabla_{\omega }$ denote the accelerometer and gyroscope bias parameters, respectively. Substituting Equations (15) and (16) into Equations (13) and (14), the bias-compensated inertial measurements can be written as follows in Equations (17) and (18):

(17) $$f_{t}^{b}={\hat{f}}_{t}^{b}-\nabla_{f},$$

(18) $$\omega_{t}^{b}={\hat{\omega }}_{t}^{b}-\nabla_{\omega },$$

Given that the SINS sampling frequency significantly exceeds that of GNSS, the pre-integration method is employed to compress inertial measurements between consecutive GNSS epochs into a single constraint factor. This approach not only reduces computational burden but also mitigates error accumulation through incremental integration, while maintaining synchronization with the GNSS observations.

Let the state vector output instants be denoted as $t_{k}$, where k=0,1,⋯ represents the time sequence indices, with a sampling interval $\Delta t=t_{k+1}-t_{k}$. At time instant $t_{k}$, the SINS measurements in the b-frame are processed via pre-integration to compute the velocity, position, and attitude increments, as expressed in Equations (19)–(21):

(19) $$\Delta v_{t_{k+1}}^{t_{k}}=\int_{t_{k}}^{t_{k+1}}(C_{b_{t}}^{b_{t_{k}}}({\hat{f}}_{t}^{b}-\nabla_{f}))dt,$$

(20) $$\Delta p_{t_{k+1}}^{t_{k}}=\int \int_{t_{k}}^{t_{k+1}}(C_{b_{t}}^{b_{t_{k}}}({\hat{f}}_{t}^{b}-\nabla_{f}))dt^{2},$$

(21) $$\Delta \phi_{t_{k+1}}^{t_{k}}=\int_{t_{k}}^{t_{k+1}}(E_{b_{t}}^{b_{t_{k}}}({\hat{\omega }}_{t}^{b}-\nabla_{\omega }))dt,$$

where $C_{b_{t}}^{b_{t_{k}}}$ and $E_{b_{t}}^{b_{t_{k}}}$ represent the rotation matrix and the angular rate matrix from the b-frame at the current time to the b-frame at time $t_{k}$, respectively. Following the SINS dead reckoning principle, the vehicle’s position, velocity, and attitude at time $t_{k+1}$ are derived as expressed in Equations (22)–(24):

(22) $$v_{t_{k+1}}^{n}=v_{t_{k}}^{n}+g^{n}\Delta t+C_{b_{t_{k}}}^{n}\Delta v_{t_{k+1}}^{t_{k}},$$

(23) $$p_{t_{k+1}}^{n}=p_{t_{k}}^{n}+v_{t_{k}}^{n}\Delta t+\frac{1}{2}g^{n}\Delta t^{2}+C_{b_{t_{k}}}^{n}\Delta p_{t_{k+1}}^{t_{k}},$$

(24) $$\phi_{t_{k+1}}^{n}=\phi_{t_{k}}^{n}+E_{b_{t_{k}}}^{n}\Delta \phi_{t_{k+1}}^{t_{k}},$$

where $g^{n}$ denotes the gravity vector, and Equations (22)–(24) collectively represent the discretized SINS state equations, which can be uniformly expressed as (25):

(25) $${\hat{x}}_{k+1}=F^{\mathrm{SINS}}\left(x_{k},\alpha_{k},z_{k}\right),$$

where $x_{k}$ represents the SINS navigation state at epoch $t_{k}$, including position, velocity, and attitude. The variable $\alpha_{k}$ denotes the inertial sensor bias states. The term $z_{k}$ corresponds to the set of high-rate inertial measurements used by the SINS mechanization to propagate the navigation state from $t_{k}$ to $t_{k+1}$, collected over the interval $\left[t_{k},t_{k+1}\right]$. The inertial measurements are available at 125 Hz, whereas the GNSS receiver provides position and velocity solutions at 1 Hz. Therefore, the state epochs $\left\{t_{k}\right\}$ are defined at the GNSS solution timestamps, and the high-rate inertial measurements between two consecutive epochs are preintegrated to generate the motion increments, as given in Equations (21)–(23). The term $F^{\mathrm{SINS}}(\cdot )$ in Equation (27) is designated as the discrete-time SINS propagation operator, which predicts the navigation state at $t_{k+1}$ from $x_{k}$, $\alpha_{k}$, and $z_{k}$. The GNSS receiver-provided position and velocity solutions are incorporated at their native timestamps by attaching the corresponding GNSS factor to the associated state node, without requiring interpolation to the SINS time scale. The predicted navigation state ${\hat{x}}_{k+1}$ is obtained from the discretized state equation, and the residual between ${\hat{x}}_{k+1}$ and the estimated state $x_{k+1}$ forms the factor-node error term minimized during optimization.

Given a predefined cost function d(⋅), the pre-integration factor node is formulated as Equation (26):

(26) $$f_{k+1}^{\mathrm{SINS}}(x_{k+1},x_{k},\alpha_{k})≜d(x_{k+1}-F^{\mathrm{SINS}}(x_{k},\alpha_{k},z_{k})),$$

### 3.3. Prior Factor and GNSS Factor

Let $f_{x}^{\mathrm{prior}}$ denote the prior factor associated with the navigation states and $f_{\alpha }^{\mathrm{prior}}$ represent the prior factor corresponding to the inertial sensor biases. These two components are jointly incorporated to construct a unified factor node, as expressed in Equation (27):

(27) $$f^{\mathrm{prior}}(x)≜d(x)=d(x-\mu_{x}),$$

where $\mu_{x}$ denotes the prior information and $\sum_{\mu }$ represents its associated covariance.

Based on the measurement data provided by GNSS, the corresponding measurement equation is formulated as follows (28):

(28) $$z_{k}^{\mathrm{GNSS}}=h^{\mathrm{GNSS}}(x_{k})+n^{\mathrm{GNSS}},$$

where $z_{k}^{\mathrm{GNSS}}$ refers to the GNSS-derived observations, including position and velocity components, $h^{\mathrm{GNSS}}(\cdot )$ denotes the GNSS measurement function with respect to state $x_{k}$, and $n^{\mathrm{GNSS}}$ represents the measurement noise. At time $t_{k}$, GNSS receives the observation and defines a new factor node $f_{k}^{\mathrm{GNSS}}(x_{k})$, which is formulated as (29):

(29) $$f_{k}^{\mathrm{GNSS}}(x_{k})=d(z_{k}^{\mathrm{GNSS}}-h^{\mathrm{GNSS}}(x_{k})),$$

### 3.4. Factor Graph Optimization

The measurement deviation of inertial devices exhibits continuous temporal variation, and the corresponding transfer function is defined in Equation (30):

(30) $${\hat{\alpha }}_{k+1}=h(\alpha_{k}),$$

The deviation factor node is formulated as shown in Equation (31):

(31) $$f_{k+1}^{\mathrm{Bias}}(\alpha_{k+1},\alpha_{k})≜d(\alpha_{k+1}-g(\alpha_{k})),$$

By simplifying Equation (31), the inertial device bias is neglected to facilitate computation. Specifically, the bias state $\alpha_{k}$ and the corresponding bias factor $f_{k+1}^{\mathrm{Bias}}\left(\cdot \right)$ are not included in the FG, and the residual influence of bias is absorbed into the process noise of the SINS propagation model. As a result, (32) and (33) are obtained:

(32) $${\hat{x}}_{k+1}=F^{\mathrm{SINS}}(x_{k},z_{k}),$$

(33) $$f_{k+1}^{\mathrm{SINS}}(x_{k+1},x_{k})≜d(x_{k+1}-F^{\mathrm{SINS}}(x_{k},z_{k}))$$

To synchronize the computational timeline of the SINS factor node with that of the GNSS factor node, Equation (33) is reformulated to yield Equation (34):

(34) $$f_{k}^{\mathrm{SINS}}(x_{k},x_{k-1})≜d(x_{k}-F^{\mathrm{SINS}}(x_{k-1},z_{k-1})),$$

Therefore, as outlined in this chapter, the measurement information from SINS and GNSS is abstracted into corresponding factor nodes, referred to as $f_{j}^{\mathrm{SINS}}$ and $f_{i}^{\mathrm{GNSS}}$, respectively. By integrating Equations (12), (29) and (34), the optimal state estimation $X_{k}^{\mathrm{MAP}}$ is constructed, and the system state is jointly optimized and solved, as expressed in Equation (35):

(35) $$X_{k}^{\mathrm{MAP}}\propto \underset{X_{k}}{\mathrm{argmin}}\left\{\begin{array}{l} ||x_{k}-F^{\mathrm{SINS}}(x_{k-1},z_{k-1}^{\mathrm{SINS}})|{|}_{\sum_{i}}^{2}\\ +||z_{k}^{\mathrm{GNSS}}-h^{\mathrm{GNSS}}(x_{k})|{|}_{\Lambda_{i}}^{2} \end{array}\right\},$$

**Figure 2:**
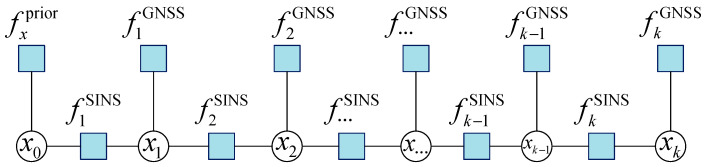
Structure of the low-cost SINS/GNSS integrated navigation system based on FG.

A FG model integrating SINS and GNSS is thereby established, as illustrated in Figure 2.

## 4. Proposed Data Forecasting Framework Using GRU-IBiLSTM

This chapter proposes a time-series prediction framework for pseudo-GNSS observations based on the GRU-IBiLSTM architecture. Section 4.1 introduces the enhanced LSTM model, focusing on its structural modifications and temporal feature extraction capabilities. Section 4.2 presents the improved BiLSTM model, emphasizing its ability to capture forward and backward dependencies in inertial sequences. Section 4.3 integrates GRU and IBiLSTM into a unified prediction framework, detailing the network design and training strategy.

### 4.1. Improved LSTM Model

As an advanced variant of RNN, LSTM integrates gating mechanisms to effectively handle sequential data.

The forget gate is responsible for discarding irrelevant information from previous time steps, as formulated in Equation (36):

(36) $$f_{n}=\mathrm{sigmoid}(W_{f}\cdot [h_{n-1},x_{n}]+b_{f}),$$

where $f_{n}$ denotes the output of the forget gate at time step n, the sigmoid function serves as the S-shaped activation function with outputs in (0,1), $W_{f}$ represents the weight matrix associated with the forget gate, $h_{n-1}$ is the hidden state at time step n−1, $x_{n}$ denotes the input vector at time step n, and $b_{f}$ corresponds to the bias term of the forget gate.

To capture the currently relevant features, the input gate is formulated as follows:

(37) $$i_{n}=\mathrm{sigmoid}(W_{i}\cdot [h_{n-1},x_{n}]+b_{i}),$$

(38) $${\tilde{C}}_{n}=\tanh(W_{c}\cdot [h_{n-1},x_{n}]+b_{c})$$

In Equations (37) and (38), $i_{n}$ represents the output of the input gate at time step n, ${\tilde{C}}_{n}$ denotes the candidate cell state at time step n, $W_{i}$ and $W_{c}$ are the respective weight matrices for the input gate and candidate memory, and $b_{i}$ and $b_{c}$ are their corresponding bias vectors.

The forget gate and input gate jointly contribute to updating the cell state, as shown in Equation (39):

(39) $$C_{n}=f_{n}⊗C_{n-1}+i_{n}⊗{\tilde{C}}_{n},$$

where $C_{n}$ and $C_{n-1}$ denote the cell states at time steps n and n−1, respectively, and ⊗ indicates the element-wise multiplication operation.

The output gate governs the generation of the current hidden state, as described in Equations (40) and (41):

(40) $$o_{n}=\mathrm{sigmoid}(W_{o}\cdot [h_{n-1},x_{n}]+b_{o}),$$

(41) $$h_{n}=o_{n}⊗\tanh(C_{n})$$

where $o_{n}$ is the output of the output gate at time step n, the tan tanh function serves as the hyperbolic tangent activation function with outputs in $\left(-1,1\right)$, $W_{o}$ is the weight matrix of the output gate, $h_{n}$ denotes the hidden state at time step n, and $b_{o}$ is the state bias term associated with the output gate.

**Figure 3:**
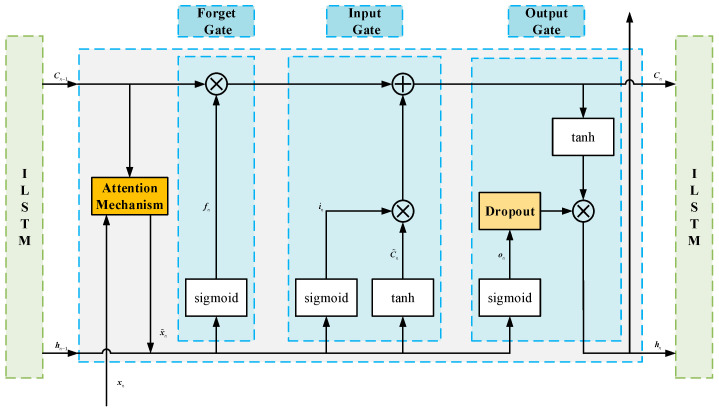
Schematic of the improved LSTM model architecture.

Given that LSTM models are prone to overfitting during training, which leads to an excessively high capacity that fits the training data and reduced generalization performance, the present study integrates a Dropout layer and an attention mechanism into the LSTM architecture, as illustrated in Figure 3.

To further enhance model robustness, the attention mechanism [35] is employed to dynamically reweight features across time steps, selectively focusing on critical temporal segments during GNSS signal interruptions. This strategy strengthens the extraction of salient features from GNSS measurements, thereby substantially improving the overall performance of the LSTM-based navigation model.

At time step n, the hidden state $h_{n-1}$ from time n−1 and the cell state $C_{n-1}$ are fed into the attention mechanism module for processing, as formulated in Equation (42):

(42) $$e_{n}^{k}=V_{a}\tanh(W_{a}\cdot [h_{n-1},C_{n-1}]+U_{a}x_{n}^{m}+b_{a}),$$

In this formulation, the attention score $e_{n}^{k}$ is computed for each input element $x_{n}^{k}$. These scores are then normalized via the softmax function to obtain the attention weights $a_{n}^{k}$, as defined in Equation (43):

(43) $$a_{n}^{k}=\frac{e^{e_{n}^{k}}}{\sum_{i=1}^{m}e^{e_{n}^{i}}},$$

The attention weights $a_{n}^{k}$ are used to weigh the corresponding input features $x_{n}^{k}$ to generate the weighted feature representations, as shown in Equation (44):

(44) $${\tilde{x}}_{n}=\left(a_{n}^{1}x_{n}^{1},a_{n}^{2}x_{n}^{2},a_{n}^{3}x_{n}^{3},\cdots ,a_{n}^{m}x_{n}^{m}\right),$$

The resulting weighted feature sequence ${\tilde{x}}_{n}$ is then fed into the LSTM unit for further processing. In this attention mechanism, $V_{a}$, $W_{a}$, and $U_{a}$ denote the weight parameter matrices associated with the module. $b_{a}$ represents the bias term.

The Dropout layer [36] functions by randomly deactivating a subset of neurons during forward propagation, thereby alleviating overfitting and enhancing the model’s generalization capability. Empirical evidence indicates that the dropout rate is typically set to [0.2,0.5], with optimal performance achieved when applied within the forward pass of gating units. In alignment with this, we incorporate the Dropout layer into the forward path of the output gate. During training, a fraction of nodes within the output gate’s weight parameter matrix is randomly deactivated. At each training iteration, a stochastic subset of nodes is selected and deactivated by zeroing their hidden parameters, while the remaining active nodes are scaled proportionally, as expressed in Equation (45):

(45) $$o_{n}=\mathrm{sigmoid}(W_{o}\cdot [h_{n-1},x_{n}]+b_{o})⊙m_{\mathrm{Dropout}}^{o_{n}},$$

where $m_{\mathrm{Dropout}}^{o_{n}}$ denotes the probability matrix that governs the deactivation process.

### 4.2. Improved BiLSTM Model

**Figure 4:**
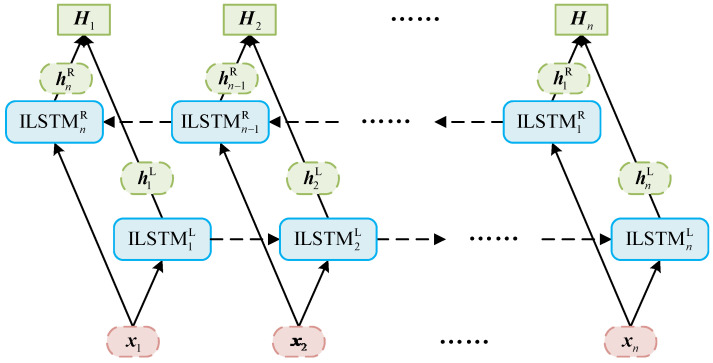
Architecture of the IBiLSTM model.

The IBiLSTM neural network comprises two ILSTM layers with an equal number of hidden units, specifically a forward ILSTM and a backward ILSTM. This bidirectional architecture enables feature extraction in both temporal directions, thereby enhancing the model’s ability to capture contextual dependencies, as illustrated in Figure 4. In this figure, $x_{1},x_{2},\cdots ,x_{n}$ denote the input vectors, which are fed into the forward network (L) in chronological order and into the backward network (R) in reverse chronological order. $h_{n}^{L}$ and $h_{n}^{R}$ correspond to the output values of the forward and backward ILSTM units at time step n, respectively. The final output $H_{n}$ is formed by concatenating the hidden states from both ILSTM units at the same time step.

### 4.3. GRU-IBiLSTM Model

The fundamental architecture of the GRU, as defined by Equations (46)–(49), exhibits structural similarities to LSTM networks, as both are RNNs that utilize gating mechanisms to regulate information flow. However, the key distinction lies in GRU’s integration of the forget and output gates into a single update gate, along with the merging of short-term and long-term memory representations. This simplified architecture has fewer trainable parameters and typically reduces per-iteration computational cost, which may improve training efficiency and lead to faster convergence in some settings. In certain application scenarios, GRU-based models can achieve performance comparable to, or sometimes better than LSTM networks.

**Figure 5:**
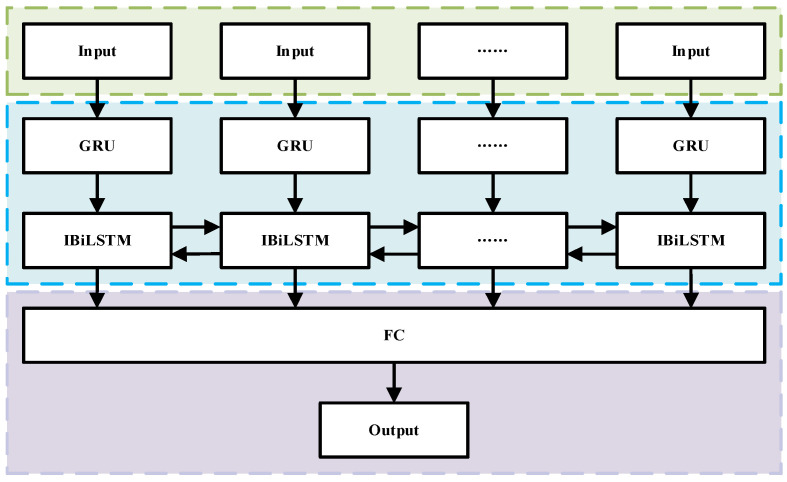
Internal architecture of the GRU-IBiLSTM model.

**Figure 6:**
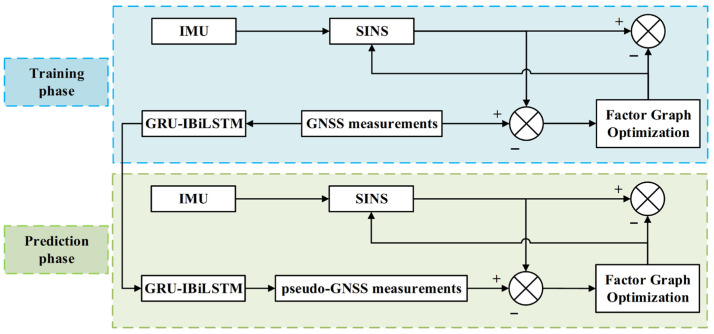
Training and prediction workflow of the proposed system.

Based on the IBiLSTM architecture and incorporating GRUs, this study proposes a GRU-IBiLSTM time-series forecasting framework. The model adopts a three-layer structure. The first layer functions as the input layer, receiving raw trajectory data. The second layer is a GRU-based encoding module that captures temporal dependencies, and the third layer comprises an IBiLSTM unit enhanced with an attention mechanism and dropout regularization, designed to improve feature learning and generalization capability. The final prediction is generated through a fully connected (FC) output layer, with the overall architecture illustrated in Figure 5. Additionally, Figure 6 presents the training and inference workflow of the proposed model:

(46) $$z_{n}=\mathrm{sigmoid}(W_{z}\cdot [h_{n-1},x_{n}]+b_{z}),$$

(47) $$r_{n}=\mathrm{sigmoid}(W_{r}\cdot [h_{n-1},x_{n}]+b_{r}),$$

(48) $${\tilde{h}}_{n}=\tanh(W_{h}\cdot [r_{n}⊗h_{n-1},x_{n}]+b_{h}),$$

(49) $$h_{n}=(1-z_{n})⊗h_{n-1}+z_{n}⊗{\tilde{h}}_{n},$$

In Equations (46)–(49), $W_{z}$, $W_{r}$, and $W_{h}$ denote the weight matrices of the update gate, reset gate, and candidate hidden state, respectively. $b_{z}$, $b_{r}$, and $b_{h}$ represent the corresponding bias vectors. $h_{n-1}$ denotes the hidden state at time step n−1, while $x_{n}$ is the input vector at time step n. $z_{n}$ and $r_{n}$ correspond to the outputs of the update gate and reset gate at time step n, and ${\tilde{h}}_{n}$ represents the candidate hidden state computed at time step n. $h_{n}$ denotes the hidden state output at time step n−1, which is also used as the input hidden state at time step n.

## 5. Experiment Results

This chapter comprehensively evaluates the performance of the proposed GRU-IBiLSTM time-series forecasting model during GNSS signal outages. Section 5.1 introduces the experimental dataset and outlines the comparative baseline methods. Section 5.2 details the simulation-based validation. Section 5.3 presents the results of onboard vehicle experiments. Section 5.4 summarizes the key findings and discusses the implications for practical deployment.

### 5.1. Experimental Data and Comparative Experiments

**Figure 7:**
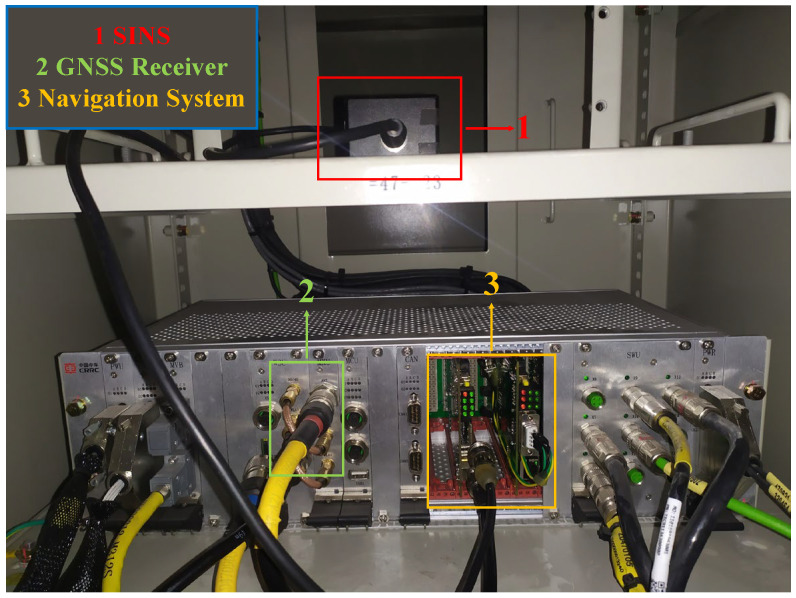
Vehicle-mounted experimental platform.

Figure 7 illustrates the vehicle-mounted experimental platform. To verify the universality and robustness of the proposed method, the model was trained and evaluated across two distinct data domains, thereby confirming that its performance is not constrained by specific environmental conditions. The simulation experiment replicated a complete operational cycle of a train, covering a total trajectory of approximately 6064.14 m over 335 s. It included representative motion states such as acceleration, slope climbing, turning, and deceleration, with the initial coordinates at (39.00854198° N, 110.4452509° E, 980.8088 m) and the final coordinates at (39.02321965° N, 110.5020469° E, 1066.4281 m). For the onboard vehicle experiment, real-world operational data were collected from a Chinese railway line, spanning approximately 2927.21 m over 275 s. The route began at (39.07855526° N, 110.57907° E, 1155.0656 m) and ended at (39.08700709° N, 110.60553° E, 1179.7364 m). All computations were executed on an Intel i9-14900HX processor (2.20 GHz) using MATLAB R2023b.

The experimental platform integrates a custom-developed navigation system consisting of a K823 GNSS receiver mounted at the rear of the lead train car and dual GNSS antennas installed on the roof of the train. The system incorporates an STIM300-based SINS operating at a sampling frequency of 125 Hz, with detailed specifications provided in Table 1. Through FGO, the system achieves complementary fusion of SINS and GNSS, effectively leveraging the strengths of both technologies to enhance overall navigation accuracy and reliability.

The evaluation procedure performs state estimation within the “East-North-Up” (“E-N-U”) coordinate frame, employing RMSE to quantify positioning accuracy along all three directions. To validate the effectiveness of the proposed GRU-IBiLSTM temporal prediction model in enhancing factor graph optimized SINS/GNSS integrated train positioning, its performance is benchmarked against four representative integration schemes:

(1) FG + GRU-IBiLSTM: A SINS/GNSS integrated train positioning method optimized using FG and enhanced by a GRU-IBiLSTM hybrid model to address GNSS signal outages and intermittent data gaps.
(2) FG + LSTM: A SINS/GNSS integrated train positioning method optimized using FG and enhanced by an LSTM model to address GNSS signal outages and intermittent data gaps.
(3) FG + BiLSTM: A SINS/GNSS integrated train positioning method optimized using FG and enhanced by a BiLSTM model to address GNSS signal outages and intermittent data gaps.
(4) FG + GRU-BiLSTM: A SINS/GNSS integrated train positioning method optimized using FG and enhanced by a GRU-BiLSTM hybrid model to address GNSS signal outages and intermittent data gaps.

For clarity, the four time-series forecasting models are denoted as follows: Model A refers to GRU-IBiLSTM, Model B to LSTM, Model C to BiLSTM, and Model D to GRU-BiLSTM.

### 5.2. Simulation Experiments

In this section, we conduct trajectory prediction simulations under normal GNSS availability and under three scenarios with complete, artificially induced GNSS outages. Section 5.2.1 evaluates the standalone performance of the four time-series forecasting models using simulated data, whereas Section 5.2.2 investigates the effectiveness of the FG-assisted fusion approach under identical experimental conditions. Under idealized conditions, the proposed method exhibits notably superior performance, thereby demonstrating its learning capability.

#### 5.2.1. Simulation Experiment—Forecasting Pseudo-GNSS Measurement Data Using Four Time-Series Forecasting Models

**Figure 8:**
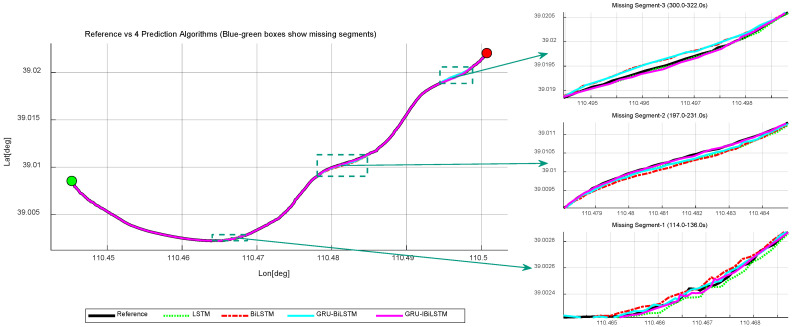
Simulated trajectories of four time-series forecasting models. The green dot indicates the train’s starting position, and the red dot indicates the train’s ending position.

To validate the proposed method, we randomly selected three time intervals from the simulation dataset to emulate GNSS signal outages. “Missing Segment-1” spans 23 s, occurring along an upward-curved road section with complete GNSS signal loss. “Missing Segment-2” lasts 35 s on an approximately linear segment, also under full GNSS interruption, while “Missing Segment-3” covers 23 s on another upward-curved trajectory, experiencing total signal deprivation. Figure 8 illustrates the trajectory comparisons between the four time-series forecasting models and the reference trajectory. As summarized in Table 2, Model A achieved RMSE values of 2.446 m, 7.709 m, and 0.139 m during “Missing Segment-1”, of 3.342 m, 8.921 m, and 0.218 m during “Missing Segment-2”, and of 5.876 m, 4.532 m, and 0.140 m during “Missing Segment-3”, thereby demonstrating its predictive performance.

**Figure 9:**
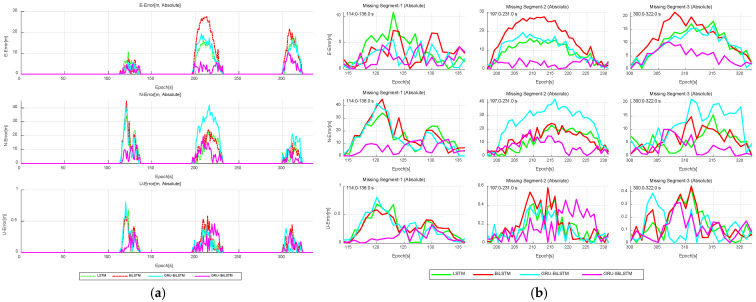
Absolute error comparison of four time-series forecasting models in “E-N-U” frame. (**a**) Overall performance. (**b**) GNSS outage scenario.

The results indicate that throughout all three GNSS outage intervals (“Missing Segment-1”, “Missing Segment-2”, and “Missing Segment-3”), Model A consistently yields lower RMSE values in both the east and north directions compared to Models B, C, and D. However, for the up direction of “Missing Segment-2”, Model A exhibits slightly lower predictive accuracy relative to Models B and D. As illustrated in Figure 9a, the positional errors of the four time-series models are compared across different directional components, while Figure 9b provides a granular breakdown of positional deviations during the outage period for each directional component, expanding upon the results shown in Figure 9a.

#### 5.2.2. Simulation Experiment—Forecasting Pseudo-GNSS Measurement Data Using FG + “Four Time-Series Forecasting Models” Method

**Figure 10:**
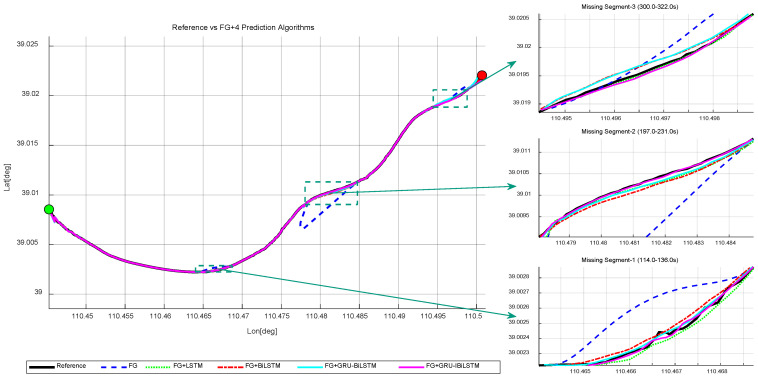
Simulated trajectories of FG-integrated time-series forecasting models. The green dot indicates the train’s starting position, and the red dot indicates the train’s ending position.

In this section, five models for SINS/GNSS integrated train positioning are evaluated. Figure 10 presents the corresponding comparison results of the FG + “four time-series forecasting models” method, and the forecasting results are summarized in Table 3. Initially, the performance of the FG-based SINS/GNSS fusion system is assessed under normal GNSS availability, yielding RMSE values of 20.501 m, 47.891 m, and 7.453 m in the three directions. To mitigate the impact of GNSS signal loss, we train the time-series forecasting models on historical GNSS measurements to generate pseudo-GNSS observations, thereby compensating for positioning errors during signal interruptions.

**Figure 11:**
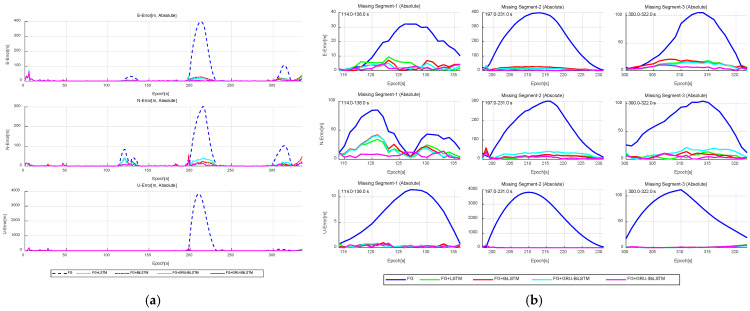

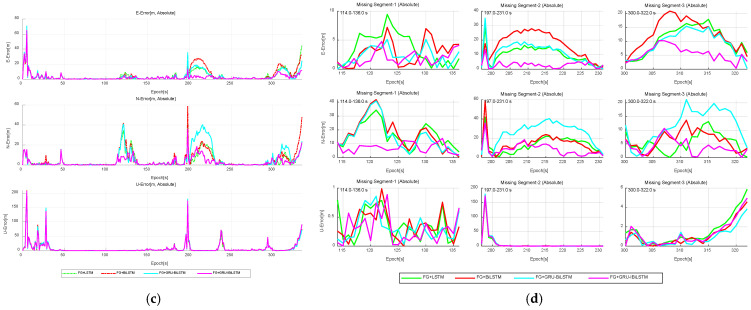
Absolute error comparison in “E-N-U” frame for FG-integrated time-series forecasting models. (**a**) Overall performance. (**b**) GNSS outage. (**c**) Without FG. (**d**) GNSS outage without FG.

As shown in Table 3, the FG + A method consistently achieves the smallest RMSE values in both the east and north directions across all three outage intervals, demonstrating superior predictive performance compared to the other three time-series models in both directions. However, in the up direction, the FG + A method exhibits slightly reduced accuracy during “Missing Segment-2” and “Missing Segment-3” relative to the two other models. This degradation is primarily attributed to the inherently higher noise levels in the up direction, where the signal-to-noise ratio is low, making it challenging for time-series forecasting methods, including FG + A and FG + C, to capture robust dynamic features in certain instances. Further visualizations in Figure 11a,c illustrate the positional errors of the FG + “four time-series forecasting models” method and the four individual time-series forecasting models across different directions. Figure 11b,d provide a detailed breakdown of directional errors during the outage periods, derived from the results in Figure 11a,c.

The simulation results confirm that during GNSS signal outages, the FG SINS/GNSS train integrated positioning system enhanced with Model A shows significantly better performance than the conventional FG SINS/GNSS integration. Notably, Model A maintains nearly perfect prediction accuracy even under the challenging conditions of “Missing Segment-1”. Quantitative analysis reveals that Model A achieves RMSE values of 2.250 m, 8.029 m, and 0.378 m along the three directions in “Missing Segment-1”, 3.841 m, 10.691 m, and 30.302 m in “Missing Segment-2”, and 6.082 m, 4.750 m, and 1.819 m in “Missing Segment-3”, and it consistently exhibits lower error magnitudes across all test segments. These findings substantiate that Model A substantially enhances positioning accuracy compared to other models in the simulation experiments. Additionally, trajectory analysis reveals notable deviations from the reference path in the navigation outputs of the baseline FG, FG + B, FG + C, and FG + D models under these demanding conditions.

### 5.3. Vehicle Experiments

**Figure 12:**
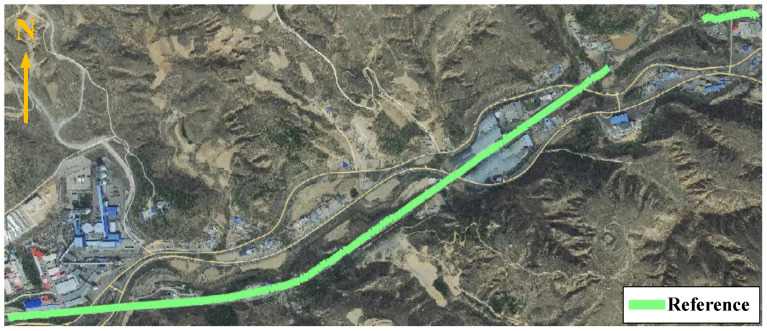
Onboard vehicle experimental line.

Similarly, this section examines three representative GNSS signal interruption scenarios, including two artificially generated segments and one naturally occurring segment collected on a railway line segment in China. Figure 12 illustrates the reference trajectory of the train, which is generated by an RTK/SINS integrated navigation solution. In open-sky environments, RTK fixed solutions are used as the reference, whereas during GNSS-denied intervals or naturally occurring signal gaps, SINS propagation is activated to provide short-term bridging, with a configurable timeout parameter for GNSS outage handling. Since no external ground truth is available within the tunnel, no error evaluation is performed for that segment. The evaluation is conducted in two stages based on real-world train operation data. Section 5.3.1 evaluates the standalone performance of the four time-series forecasting models using real-world train operation data. Section 5.3.2 investigates the fusion method enhanced by FG, also based on real-world data, in order to demonstrate its robustness under GNSS-denied conditions. Experimental results confirm that the proposed method delivers competitive performance, even in environments characterized by high noise and frequent signal degradation.

#### 5.3.1. Onboard Vehicle Experiment—Forecasting Pseudo-GNSS Measurement Data Using Four Time-Series Forecasting Models

“Missing Segment-X” lasted for 39 s and occurred along an upward-curved track section experiencing complete GNSS signal interruption. “Missing Segment-Y”, also spanning 39 s, took place on a straight track segment under full GNSS signal loss. “Missing Segment-Z”, likewise persisting for 39 s, corresponded to a downward-curved section where the train entered a tunnel, resulting in another complete GNSS outage. To predict GNSS measurements during the three interruption periods, Model A was utilized, with Models B, C, and D selected for comparative performance evaluation.

**Figure 13:**
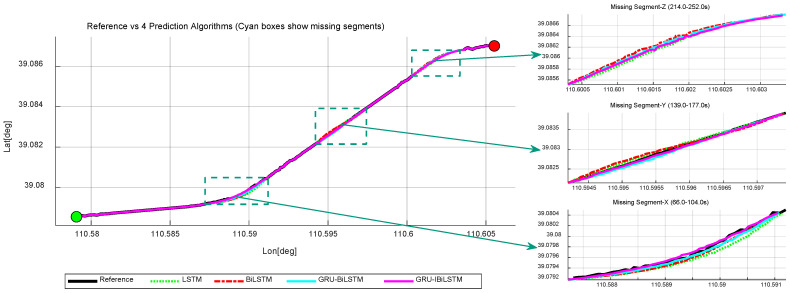
Vehicle-mounted experimental results. Trajectory comparison of four time-series forecasting models. The green dot indicates the train’s starting position, and the red dot indicates the train’s ending position.

**Figure 14:**
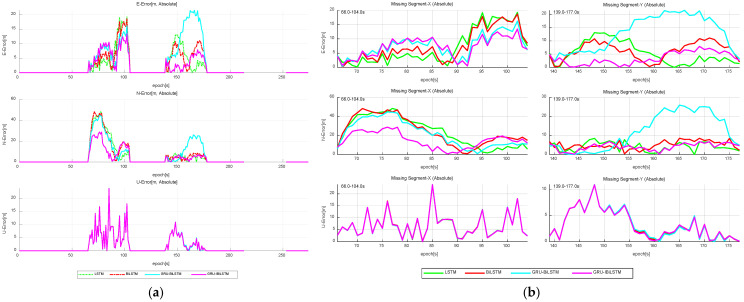
Absolute error comparison in “E-N-U” frame for four time-series forecasting models in vehicle experiments. (**a**) Overall performance. (**b**) GNSS outage scenario.

Figure 13 compares the predicted trajectories from the four time-series forecasting models with the reference trajectory, and the forecasting results for specific railway segments are summarized in Table 4. During “Missing Segment-X”, Model A achieved RMSE values of 7.904 m, 16.986 m, and 8.512 m in the east, north, and up directions, respectively. In “Missing Segment-Y”, the RMSE values were further reduced to 4.182 m, 4.491 m, and 4.311 m for the same directional components. Notably, during “Missing Segment-Z”, where GNSS measurements were entirely unavailable as the train passed through the tunnel, pseudo-GNSS predictions generated by Model A were employed to compensate for the signal loss. Across both GNSS-denied intervals (“Missing Segment-X” and “Missing Segment-Y”), Model A consistently yielded lower RMSE values in all three directional components compared to Models B, C, and D. Furthermore, as illustrated in Figure 14, Model A outperformed the other three models in prediction accuracy across the east, north, and up directions, underscoring its enhanced robustness. Figure 14a depicts the positional errors of the four time-series forecasting models in different directions, while Figure 14b provides a refined visualization of the directional deviations during the outage periods, derived from the results in Figure 14a.

#### 5.3.2. Onboard Vehicle Experiment—Forecasting Pseudo-GNSS Measurement Data Using FG + “Four Time-Series Forecasting Models” Method

**Figure 15:**
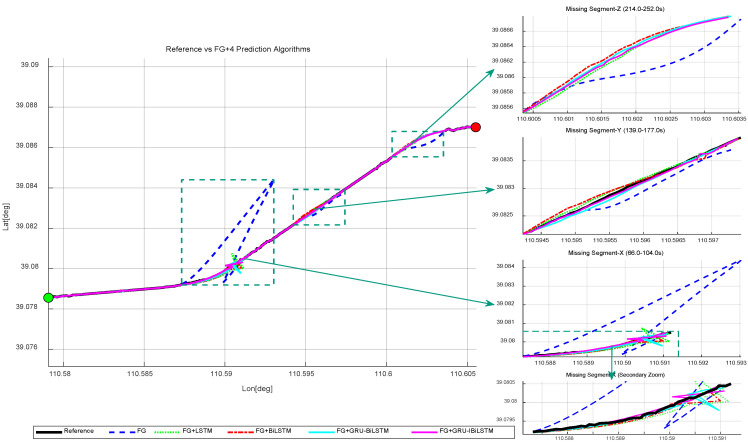
Trajectory results of FG-integrated time-series forecasting models in vehicle experiments. The green dot indicates the train’s starting position, and the red dot indicates the train’s ending position.

**Figure 16:**
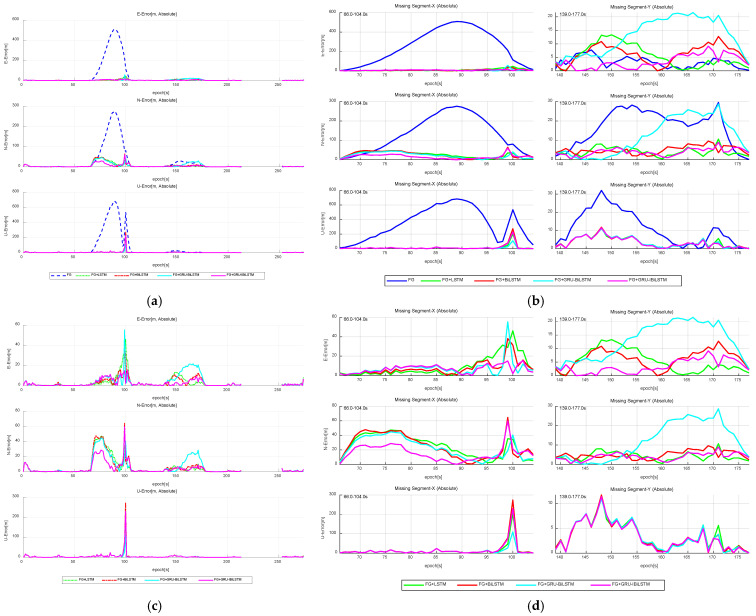
Absolute error comparison in “E-N-U” frame for FG-integrated time-series forecasting models in vehicle experiments. (**a**) Overall performance. (**b**) GNSS outage scenario. (**c**) Without FG. (**d**) GNSS outage without FG.

Similarly, this section provides a comparative evaluation of five SINS/GNSS integrated positioning configurations, with the trajectory comparisons shown in Figure 15 and the quantitative results summarized in Table 5. Figure 16a,c illustrate the directional positional errors of the FG + “four time-series forecasting models” method along different directions. The results demonstrate that the FG + A configuration consistently achieves lower RMSE values during GNSS signal interruptions under challenging conditions, outperforming alternative configurations, including FG + B, FG + C, and FG + D. Although the FG + A method exhibits slightly elevated errors in the up direction compared to FG + D and marginally underperforms FG + B during the initial “Missing Segment-X” phase, it still reduces RMSE values to 8.012 m, 19.146 m, and 39.693 m along the east, north, and up directions, respectively, which represents a substantial improvement over the baseline FG method. Notably, the baseline FG configuration fails to retain original GNSS measurements during the subsequent “Missing Segment-Y” phase, whereas the proposed method achieves superior RMSE values of 4.406 m in the north direction and 4.343 m in the up direction, consistently outperforming conventional approaches. These findings confirm the positioning accuracy advantage of the FG + A model. A more detailed analysis of directional error distributions during GNSS outages is provided in Figure 16b,d, based on the data presented in Figure 16a,c.

### 5.4. Chapter Summary

This chapter evaluated the localization performance of the proposed FG + A method through comprehensive experiments. The results demonstrate that the method effectively balances cost-efficiency with high-accuracy train localization, showing strong robustness and generalization across diverse railway environments. It consistently outperforms other approaches in the East and North components. Although the improvement in the Up direction is less significant, this is primarily due to the higher noise and uncertainty inherent in the GNSS vertical observations. Despite this limitation, the framework maintains stable overall performance and exhibits notable resilience to GNSS signal degradation, highlighting its practical value as a reliable solution for low-cost, high-accuracy train localization.

## 6. Conclusions

To address train positioning failures caused by GNSS signal interruptions in complex environments, this paper proposes a GNSS prediction method based on a GRU-IBiLSTM network. The proposed model generates pseudo-GNSS observations, enabling low-cost SINS/GNSS integrated train positioning systems to maintain high accuracy during GNSS outages. Building on the proposed prediction model, the study further develops the probabilistic FG framework tailored for SINS/GNSS integrated positioning, which enables effective fusion of pseudo-GNSS observations and SINS estimates during signal interruptions. Compared with traditional single-model prediction approaches, the proposed hybrid architecture exhibits enhanced robustness and temporal modeling capability, as it leverages the complementary strengths of GRU and IBiLSTM networks. Integrating the FG framework with the GRU-IBiLSTM temporal prediction model yields significantly higher positioning accuracy, which is attributed to the synergistic advantages of global optimization, plug-and-play modularity, deep temporal feature extraction, and bidirectional utilization of historical information. Despite the strong performance of the proposed method in horizontal positioning accuracy, the current implementation of the proposed method does not yet satisfy stringent real-time constraints. Accordingly, future work will prioritize real-time operation and will further explore universal temporal prediction models with cross-scenario generalization capabilities and incorporate train-specific motion constraints to further improve vertical accuracy, thereby enhancing the method’s adaptability to diverse railway lines and varying operational environments.

## Figures and Tables

**Table 1:** Key performance indicators of the sensors.

sensor	Parameter	Index
Low-cost SINS	Gyro drift	5°/hr
	Gyro random noise	0.1°/√hr
	Accelerometer bias	2 mg
	Accelerometer noise	0.07 m/s/√hr
	Data update rate	125 Hz
GNSS	Velocity accuracy	0.02 m/s
	Position accuracy	H ≤ 1.5 m, V ≤ 3 m
	Data update rate	1 Hz

**Table 2:** Simulation experiment—RMSE of four time-series forecasting models in the “E-N-U” directions.

Period	Directions	A (m)	B (m)	C (m)	D (m)
Missing Segment-1	E	2.446	4.503	3.815	2.950
	N	7.709	18.193	20.572	18.673
	U	0.139	0.341	0.328	0.346
Missing Segment-2	E	3.342	10.525	18.838	12.194
	N	8.921	13.644	14.284	27.416
	U	0.218	0.203	0.249	0.186
Missing Segment-3	E	5.876	11.192	3.325	10.269
	N	4.532	6.765	7.501	12.952
	U	0.140	0.186	0.194	0.166

**Table 3:** Simulation experiment—RMSE of FG + “four time-series forecasting models” method in the “E-N-U” directions.

Period	Directions	FG (m)	FG + A (m)	FG + B (m)	FG + C (m)	FG + D (m)
Missing Segment-1	E	20.501	2.250	4.401	3.575	2.707
	N	47.891	8.029	18.315	20.385	18.821
	U	7.453	0.378	0.453	0.427	0.396
Missing Segment-2	E	260.669	3.841	11.577	18.944	13.433
	N	189.301	10.691	15.540	17.331	28.000
	U	2447.313	30.302	28.672	29.694	31.537
Missing Segment-3	E	62.436	6.082	11.595	13.697	10.230
	N	72.129	4.750	6.539	7.109	13.092
	U	74.578	1.819	2.156	1.689	1.381

**Table 4:** Vehicle experiment-RMSE of four time-series forecasting models in the “E-N-U” directions.

Period	Directions	A (m)	B (m)	C (m)	D (m)
Missing Segment-X	E	7.904	9.439	9.245	8.475
	N	16.986	28.272	28.614	25.573
	U	8.512	8.521	8.533	8.519
Missing Segment-Y	E	4.182	6.738	7.026	14.455
	N	4.491	4.708	5.506	15.155
	U	4.311	4.366	4.346	4.371

**Table 5:** Vehicle experiment—RMSE of FG + “four time-series forecasting models” method in the “E-N-U” directions.

Period	Directions	FG (m)	FG + A (m)	FG + B (m)	FG + C (m)	FG + D (m)
Missing Segment-X	E	307.043	8.012	14.057	10.609	11.270
	N	160.910	19.146	29.233	29.934	26.294
	U	409.939	39.693	34.869	46.657	19.671
Missing Segment-Y	E	3.753	4.197	6.687	6.989	14.440
	N	19.071	4.406	4.575	5.386	15.127
	U	14.373	4.343	4.500	4.422	4.433

## Data Availability

The data presented in this study are available on request from the corresponding author due to restrictions arising from legal and confidentiality agreements signed with the data provider. It should also be noted that this study was conducted under a data confidentiality agreement with China State Railway Group Co., Ltd.
